# Effects of Organized Physical Activity on Selected Health Indices among Women Older than 55 Years

**DOI:** 10.1155/2015/625032

**Published:** 2015-05-27

**Authors:** Piotr Zmijewski, Krzysztof Mazurek, Ewa Kozdron, Piotr Szczypiorski, Agata Frysztak

**Affiliations:** ^1^Department of Physiology, Institute of Sport, 01-982 Warsaw, Poland; ^2^Department of Sports Medicine, Jozef Pilsudski University of Physical Education in Warsaw, 00-968 Warsaw, Poland; ^3^Department of Tourism and Recreation, Jozef Pilsudski University of Physical Education in Warsaw, 00-968 Warsaw, Poland

## Abstract

The main aim of this study was to determine health benefits among women older than 55 years who participated in organized, group-based physical activity (OPA). Thirty-five women aged 65.0 ± 7.3 years volunteered for this study. The classical and nonclassical cardiovascular (CVD) risk factors were measured before and after a 2-week OPA camp in a remote location and 3 months of OPA. Self-guided physical activity was analyzed 18 months after OPA. Two-week effects included significant decreases in body mass index, waist and hip circumferences, resting systolic and diastolic blood pressure (BP) and resting heart rate, improved exercise capacity (EC), improved low-density lipoprotein and high-density lipoprotein (HDL-C), cholesterol, and other atherogenic lipid indices (ALI), and a reduction in 10-year estimated risk of death from CVD. Three-month effects included a further decrease in systolic BP, improvements in EC and HDL-C, and maintenance of lower levels of ALI, as well as lower CVD risk. The implementation of the OPA programme had a positive impact on somatic features, exercise capacity, biochemical indices, and risk for death from CVD. The presented programme can be regarded as an effective element of primary prevention of cardiovascular diseases among women older than 55 years.

## 1. Introduction

It is expected that the number of elderly people (>60 years old) in Poland will increase to 26.7% of the total population by 2035, primarily due to increasing life expectancy [1]. A similar trend is observed worldwide, especially in developed countries. The process of aging results in physical fitness reduction (especially strength, endurance, agility, and flexibility), which leads to functional problems in daily activities and difficulties in normal functioning of the elderly [2, 3]. Physical fitness appears to be a significant element of quality of life [4]. Moreover, regular physical activity and physical fitness were also established as independent predictors for cardiovascular diseases (CVD) [5].

It is well established that systematic physical activity is associated with health benefits; it reduces risk for all-cause mortality, hypertension, coronary heart disease, obesity, diabetes, breast cancer, and osteoporosis, and it improves the mental health and quality of life of the elderly [6–8]. Despite that, the level of physical activity is insufficient in many societies. Physical inactivity is believed to be one of the most important public health problems in the 21st century [9].

In Poland, similarly to other developing and developed countries, low levels of physical activity are noted, both in children and in adults [10, 11]. Furthermore, relatively high prevalence of metabolic diseases, obesity, and hypertension is noted in adults, accompanied by a generally low level of awareness of these diseases [12–14].

Retirement seems to have upmost importance for adult behaviours and is perceived as a key transition which impacts physical and social activities [15]. In the period of retirement, physical activity associated with work-related transportation is reduced. Moreover, this decrease in active transportation is not compensated for by additional recreational physical activity [15]. Therefore, the retirement transition could be regarded as a period of good opportunity for intervention aimed at encouraging and developing behaviours of regular physical activity. Effective approaches to help preolder and older people maintain a healthy and active lifestyle as well as evaluation of effectiveness of physical activity intervention programmes adopted to specific communities are needed. Therefore, the aim of this study was to determine health benefits among women older than 55 years who participated in an organized, group-based physical activity (OPA) programme.

## 2. Materials and Methods

### 2.1. Subjects and Experimental Design

Thirty-five Caucasian women aged 65.0 ± 7.3 years volunteered for this study. Subjects were recruited through advertisements in an academic community for participation.

The criteria to be included in the study were age >55 years, being female, no health contraindications (confirmed by medical examination), and consent for participation in the whole study (exercises and examinations). Subjects' characteristics are presented in Table 1.

The subjects were instructed to maintain their usual nutritional habits throughout the training period; no nutritional intervention was introduced. Before starting the study, all participants received a complete explanation of the purpose, risks, and procedures of the investigation and provided written informed consent. The investigation was approved by the Ethics and Research Committee of the Jozef Pilsudski University of Physical Education in Warsaw, Poland.

### 2.2. Study Design

Participants were physically tested on three occasions. The first one took place before the Active Leisure Time Programme (ALTP), pretraining outcomes, treated as baseline characteristics. The second one was performed within two days after ALTP, the first posttraining outcome, regarded as short-term effects. The third part of measurements was conducted within two days after the 3-month Prevent Falls in the Elderly Programme, posttraining outcome, treated as intermediate-term effects. Additionally, one was performed correspondently with a specific questionnaire to analyse the level of self-guided physical activity 18 months after ALTP (long-term effects). The study organization was summarized in Table 2.

### 2.3. Exercise Protocol

#### 2.3.1. Active Leisure Time Programme (ALTP)

Firstly, participants followed a multicomponent exercise training programme in a remote location (a municipal sports centre in Kozienice, Poland). ALTP includes two weeks of daily physical activities, performed 3 times per day, supplemented by additional social, educational, and motivational activities. Exercise activities include general fitness classes and yoga (with the main goal of muscle strength and body balance), Nordic walking, and aqua fitness. In total, 39 physical activities were performed within two weeks. Exercise intensity was controlled by heart rate, which ranged within 40–60% of maximal heart rate. Simultaneously, subjects controlled exercise intensity by ratings of perceived exertion (RPE) using the 6–20 Borg Scale [16]. Participants were instructed that RPE should range between 8 and 15 points. 20% of exercise was ranked below 9 points, 70% was ranked within 10–12 points, and 10% was assessed within 13–15 points. The sessions were conducted by a physical education instructor with specialization in training older adults and consisted of three parts: (1) general warm-up activity, calisthenics, and stretching exercises at the start of sessions; (2) aerobic exercises; and (3) cool-down activities. Each session lasted 40–75 minutes, making a total of 3-4 hours per day. The programme was previously described in detail by Kozdron [17].

#### 2.3.2. Prevent Falls in the Elderly Programme (PFEP)

A 3-month programme of general fitness exercise (gymnastics, body balance, and muscle strength) was conducted with the main purpose to prevent falls in the elderly. Independently of participating in programmed activities, informational sessions aimed to encourage undertaking self-guided physical activities were performed. Regular activities were conducted in 2 sessions per week.

#### 2.3.3. Self-Guided Physical Activities

At the end of the ALTP, the participants were asked to participate in self-guded physical activities. This was an 18-month period; physical activities were not limited, not prescribed, and not controlled.

### 2.4. Measurements

Prior to the beginning of organized, group-based physical activity (OPA), subjects were questioned about their health status by a medical doctor. Subjects were examined two days prior to the beginning of the ALTP, the following day after the ALTP, and the next day after the Prevent Falls in the Elderly Programme, and the data concerning physical activity level were collected 18 months after the ALTP.

### 2.5. Anthropometrics

Somatic measures were obtained while subjects were dressed in light clothing without shoes. Height and body mass were recorded using a portable stadiometer and balance weighting scales, respectively. Body mass index (BMI) was calculated using the standard formula: mass (kg)/height^2^ (m). Waist circumference was measured around the abdomen at the level of the umbilicus. Hip circumference was measured at the level of the maximum extension of the buttocks posteriorly in a horizontal plane. WHR was calculated as waist circumference (cm) divided by hip circumference (cm), while WHtR was calculated as waist circumference (cm) divided by height (cm) [18]. Percent body fat was measured using bioelectrical impedance (Tanita, Model BC-545 Tanita, Japan) performed during 8.00–10.00 a.m. In accordance with the manufacturer's instructions, the subjects stood on the metal footpads in bare feet, and fat mass (%) was determined using the prediction equations supplied by the manufacturer. Fat mass (kg) and fat-free mass (%) were subsequently calculated from body weight using the measured fat mass (%). Before the measurements subjects had fasted for 12 h and not exercised for at least 12 h.

### 2.6. Physical Fitness

Aerobic capacity and exercise tolerance were measured using an incremental exercise stress test performed on an electrically braked bicycle ergometer. The test started with a 3 min warm-up at a workload of 10 W and continued with 20 W increments every two minutes until reaching a limit of 75% maximal heart rate (HRmax). HRmax was estimated with the following equation: HRmax = 220 − age (years). At the end of test the participants were asked to rate their perceived exertion using 6–20 Borg scale. The subjects were informed about the test protocol beforehand.

Electrocardiography (ECG) was recorded throughout the exercise test (CASE Value, GE Healthcare). Blood pressure was measured every 2 min during the test using a sphygmomanometer by the Korotkow method. Heart rate was recorded via ECG. Oxygen consumption was estimated using the equation of Storer et al. [19]. Some serious syndromes during or after the exercise tests gave reasons to exclude a candidate from the exercise activity programme. The following outcomes were included into further analysis: time of exercise in protocol, maximal tolerated load in exercise protocol expressed in Watt, maximal tolerated load in exercise protocol expressed in metabolic equivalent units, maximal oxygen consumption, and rate of perceived exertion at the end of exercise protocol.

Physical fitness level was assessed with criteria of physical fitness proposed by Hakola et al. [20].

### 2.7. Blood Profile

Blood for biochemical assays was withdrawn from the antecubital vein. The pre- and posttraining venous blood samples were obtained from the participants between 8:00 and 10:00 a.m. after a 12-hour overnight fast. Routine biochemical methods were used for the determination of TC, TG, HDL-C, glucose, and hsCRP. LDL-C was calculated using the formula of Friedewald. All measurements were carried out at the biochemistry laboratory of the University of Physical Education in Warsaw, Poland.

### 2.8. Cardiac and Pulmonary Indices

Blood pressure and heart rate recorded at rest by an experienced physician, using a standard mercury sphygmomanometer. The pulmonary outcomes were forced vital capacity (FVC), forced expiratory volume in one second (FEV1), and Tiffeneau index, which is the ratio between forced expiratory volume in one second and forced vital capacity (FEV1/FVC). Three manoeuvres were performed in order to obtain only the best values for the analysis. The measurements were conducted using an Easy-One 2001 spirometer (ndd Medical Technologies, Zürich, Switzerland). The values were expressed in absolute terms and as a percentage of the theoretical value for individuals of the same age, weight, and height in the reference population.

### 2.9. Statistics

Descriptive measures were calculated for all variables. Data were verified for normality of distribution with the Kolmogorov-Smirnov test. Variables that did not meet the assumption of normality were analysed with nonparametric statistics.

One-way analysis of variance or the Kruskal-Wallis test was used to compare means of continuous variables before and after the training periods. Bonferroni's test or the Wilcoxon rank test was used for post hoc analysis. The statistical level was set at *P* ≤ 0.05. Statistical tests were performed with SPSS software (v. 17; SPSS Inc.)

## 3. Results

**Figure 1:**
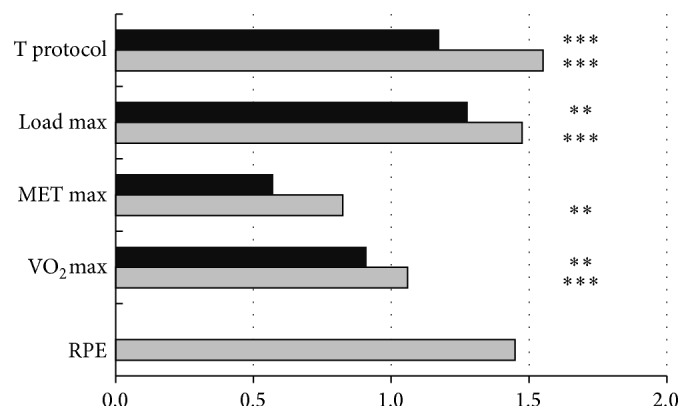
Rate of changes of physical fitness indices after Active Leisure Time Programme (black bars) and Prevent Falls in the Elderly Programme (grey bars), compared to baseline. Note: values are mean normalised outcomes. T protocol: time of exercise in protocol; load max: maximal tolerated load in exercise protocol expressed in Watt; MET max: maximal tolerated load in exercise protocol expressed in metabolic equivalent units; VO_2_max: maximal oxygen consumption; RPE: rate of perceived exertion at the end of exercise protocol. Levels of statistical significance: ^*∗∗*^ *P* < 0.01, ^*∗∗∗*^ *P* < 0.001.

As effect of 2-week and 3-month exercise intervention main physical fitness outcomes were significantly improved (Figure 1). Comparing to baseline, the time of exercise in protocol was significantly increased at first follow-up (*P* < 0.001) and at second follow-up measurements (*P* < 0.001). Also, the maximal tolerated load in exercise protocol was greater at first (*P* < 0.01) and second follow-up measurements (*P* < 0.001) than at baseline. These changes were reflected by improvements in estimated VO_2_max (*P* < 0.01 and *P* < 0.001, resp.). The rate of perceived exertion at the end of exercise protocol remained similar to baseline after 2-week exercise intervention; after 3-month of exercise participants' rate of perceived exertion increased insignificantly.

**Figure 2:**
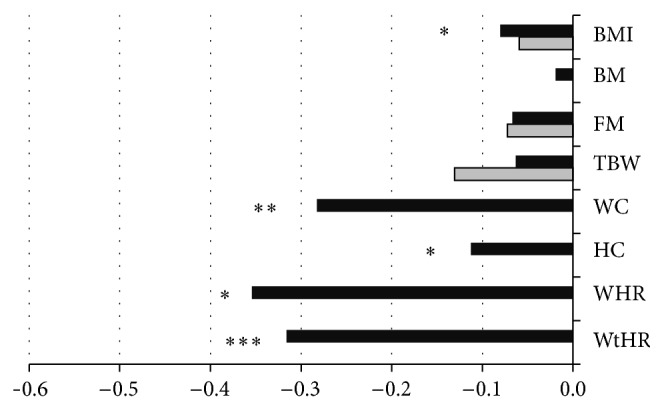
Rate of changes of somatic variables after Active Leisure Time Programme (black bars) and Prevent Falls in the Elderly Programme (grey bars), compared to baseline outcomes. Note: values are mean normalised outcomes. BMI: body mass index; BM: body mass; FM: fat mass; TBW: total body water; WC: waist circumference; HC: hips circumference; WHR: waist to hip ratio; WtHR: waist to height ratio. Levels of statistical significance: ^*∗*^ *P* < 0.05, ^*∗∗*^ *P* < 0.01, and ^*∗∗∗*^ *P* < 0.001.

Body mass and fat mass did not change significantly after 2-week and 3-month exercise intervention (Figure 2). BMI was significantly lower after ALTP (*P* < 0.05), but after PFEP the decrease was statistically insignificant. After ALTP waist and hip circumferences were statistically decreased, when compared to baseline (*P* < 0.01 and *P* < 0.05, resp.). Similarly, WHR and WHtR indices were also improved after ALTP (*P* < 0.05 and *P* < 0.001, resp.).

**Figure 3:**
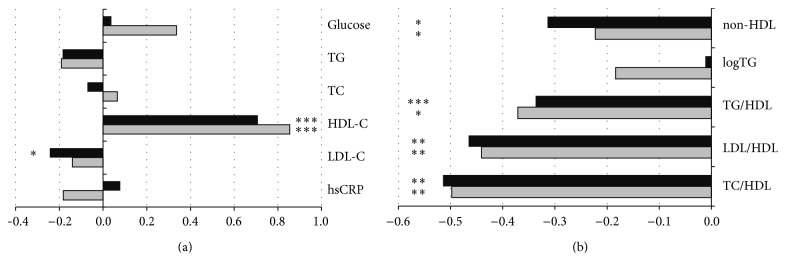
(a-b) Rate of changes of blood biochemical indices after Active Leisure Time Programme (black bars) and Prevent Falls in the Elderly Programme (grey bars), compared to baseline. Note: values are mean normalised outcomes. TG: triglycerides; TC: total cholesterol; HDL-C: cholesterol high density lipoprotein; LDL-C: cholesterol low density lipoprotein; hsCRP: high sensitive C-reactive protein. Levels of statistical significance: ^*∗*^ *P* < 0.05, ^*∗∗*^ *P* < 0.01, and ^*∗∗∗*^ *P* < 0.001.

Fasting glucose, TG, TC, and hsCRP concentrations did not change significantly after 2-week and 3-month interventions (Figure 3). HDL-C increased significantly after ALTP and PFEP, when compared to baseline (*P* < 0.001 and *P* < 0.001, resp.). LDL-C lowered significantly after 2-week exercise intervention (*P* < 0.05), but changes were not maintained after 3 months. The other lipid atherogenic indices (non-HDL, TG : HDL, LDL : HDL, and TC : HDL) decreased significantly after ALTP (*P* < 0.05, *P* < 0.001, *P* < 0.01, and *P* < 0.01, resp.) and remained decreased after PFEP, when compared to baseline (*P* < 0.05, *P* < 0.05, *P* < 0.01, and *P* < 0.01, resp.). Only the values of logTG did not change within whole study.

**Figure 4:**
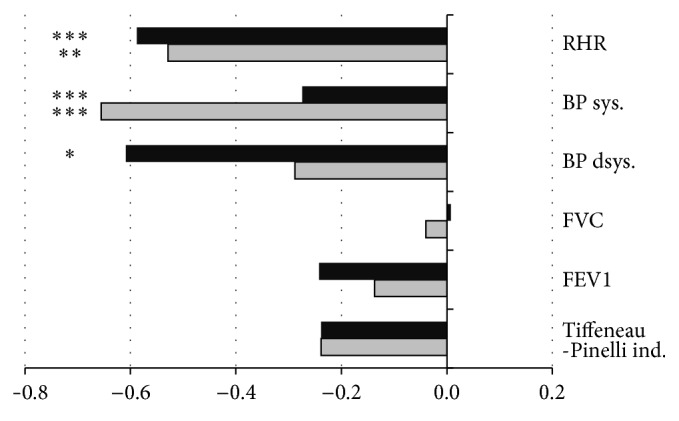
Rate of changes of cardiac and spirometric indices after Active Leisure Time Programme (black bars) and Prevent Falls in the Elderly Programme (grey bars), compared to baseline. Note: values are mean normalised outcomes. RHR: heart rate at rest; BP sys.: systolic blood pressure; BP dsys.: diastolic blood pressure; FVC: forced vital capacity; FEV1: forced expiratory volume in 1 second; FEV1/FVC: Tiffeneau-Pinelli index. Levels of statistical significance: ^*∗*^ *P* < 0.05, ^*∗∗*^ *P* < 0.01, and ^*∗∗∗*^ *P* < 0.001.

The significant decreases in heart rate at rest (RHR), resting systolic (BP sys.), and resting diastolic blood pressure (BP dsys.) were noted after 2-week exercise intervention (*P* < 0.001, *P* < 0.001, and *P* < 0.05, resp.), when compared to baseline. After 3-month intervention RHR and BP sys. were lower than at baseline (*P* < 0.01 and *P* < 0.001), but BP dsys. was not statistically improved when compared to baseline (Figure 4). The pulmonary indices like FVC, FEV1, and Tiffeneau index did not change significantly within the study.

## 4. Discussion

The main aim of this study was to determine the health benefits gained by women older than 55 years, who participated in the organized physical activity programme. This study examined the effects of specific physical activity programmes on the magnitude of changes in selected somatic, biochemical, spirometric, cardiac, and physical fitness indices. Generally, this research confirms the assumption that regular physical activity allows one to gain health benefits as a result of structured short- and intermediate-term interventions [21, 22]. This research could be regarded as interesting as it aimed to evaluate a specific physical activity programme [17], intended for preolder women in the retirement period, in the context of both health and physical fitness.

In this study, participation in a physical activity programme resulted in increase of physical capacity after only 2 weeks of physical exercise and subsequently its maintenance (VO_2_max, maximal load) or further increase (time of exercise in testing protocol) within 3 months. Finally, the capability to continue exercise and to exercise with higher load was increased significantly (*P* < 0.001 and *P* < 0.001, resp.). The changes in estimated VO_2_max (18.6%; *P* < 0.05) were relatively high. Similar results could be expected following a 16-week period of increased physical activity and further increase is possible after 20 weeks or intervention involving interval training [23–25]. These findings could be important for participants' health as low physical fitness is a significant precursor of premature cardiovascular and noncardiovascular mortality [26]. Furthermore, in the first stage of the analysed programme, adaption to moderate-to-vigorous intensity of exercise was obtained, which is included in current recommendations [27].

Despite beneficial changes in physical capacity, no positive changes were noted for spirometric indices. Nevertheless, baseline indices of FVC and FEV1 were relatively high, respectively, 114% and 106% of reference values. Maintaining high spirometric indices in the elderly is desirable as it was established that, with the decrease in pulmonary function, the risk of cardiovascular events significantly increases [28].

The findings of this study are in contrast with results from other studies that did find a lowering of BMI and fat mass effect in elderly subjects after aerobic training. BMI significantly decreased in only 2 weeks of ALTP, but this effect, similarly as for body and fat mass decrease, was not maintained within the whole period of observation. Findings from the other studies suggest that, as a result of physical activity of moderate intensity (>60% VO_2_max), the body mass could be decreased by 1–4% within 2–9 months, mainly due to a decrease in body fat [29]. However, other investigators also did not note a significant decrease in body mass in physically active elderly people. Manini et al. found that changes in energy expenditure for activity is not associated with changes in body mass composition in older women [30]. A plausible explanation for not finding positive changes in body composition after 3 months of physical training may be the relatively low contribution of exercise of moderate-to-vigorous intensity (13–15 points on the Borg Scale; approximately 10%), despite quite high exercise volume. Nevertheless, significant reductions of waist (*P* < 0.05) and hip circumferences (*P* < 0.05) as well as WHR (*P* < 0.02) were noted. Both waist circumferences and WHR are considered as independent predictors of cardiovascular diseases (CVD) and risk of all-cause mortality [31, 32]. In wide, randomized clinical trials health effects such as positive changes in lipid profile, improved cardiorespiratory fitness, and a hypotensive effect of increased physical activity were noted in older people, even when body mass was not reduced [33]. The obtained results support hypothesis that weight loss is not mandatory for exercise-induced effects on health indices [34].

The results of this study support findings that regular physical activity has a positive impact on lipid profile. After only two weeks of a multicomponent exercise training programme in a remote location a significant increase in HDL-C concentration (*P* < 0.001) and decrease in LDL-C concentration (*P* < 0.05) were noted. Within the next three months of systematic physical activity in the Prevent Falls in the Elderly Programme, further but insignificant positive changes in HDL-C were observed, and LDL-C insignificantly decreased. Within the whole period of increased physical activity, HDL-C improved permanently, while LDL-C did not change. These results are consistent with results of earlier studies. Findings of cross-sectional studies conducted among the elderly showed that total physical activity (involving work related PA, leisure-time, and recreation PA) had a significant, positive correlation with HDL-C concentration, but no significant association was found for LDL-C and triglycerides [35]. It was determined that mean increase in HDL-C as a result of exercise intervention lasting at least 8 weeks was 2.53 mg/dL. Moreover, favourable changes occur when energy expenditure for physical activity exceeds 900 kcal per week and further increase in physical activity can bring additional health benefits [36].

It was revealed that only the concentration of LDL-C among other lipids and biomarkers of myocardial infarction does not change significantly in relation to increasing physical activity intensity level [37]. Nevertheless, a large meta-analysis showed that it is possible to reduce the concentration of LDL-C by about 3% (i.e., −4.2 ± 1.1 mg/dL) as a result of at least an 8-week period of increased physical activity [38]. In the present study, after 3 months of exercise intervention, a decrease of LDL-C by about 5% was noted, but it was not statistically significant. Mean change in TG after 3 months of physical training was about 8%, but also statistically insignificant. Lack of positive changes in TG as a result of exercise training was also observed in studies aimed at determining the effectiveness of walking among adult women [39]. On the other hand, a number of studies have demonstrated the positive effect of exercise training in reduction of TG concentration, and its effectiveness depends not only on the nature of the exercise (kind and intensity), but mainly on the amount of exercise. It was found that the most favourable changes in lipid profile are achieved when the highest amount of weekly exercise, with minimal weight change, occurred [40].

In the current study, persistent and significant changes in the concentrations of total cholesterol and TG were not found. This is consistent with many other exercise-based interventional studies, but beneficial changes in LDL as a result of increased physical activity could be achieved [41]. However, contrary to the lack of beneficial changes in LDL, TC, and TG after ALTP and PFEP, in the current investigation, lipid atherogenic indices (non-HDL, TC : HDL, LDL : HDL, and TG : HDL) changed significantly in favourable directions, which at least partially confirms the antiatherogenic effects of this intervention.

One of the most important health effects for the elderly observed in this study was a reduction in resting blood pressure by an average of 8.5% and 3.1% for systolic and diastolic within 3 months. These results are in accordance with previous research that found a postexercise hypotensive effect after intervention based on a different kind of aerobic exercise [42, 43].

At 18 months following participation in programmes of organized physical activity only every seventh person was not physically active. More than 2/3 of participants continued engagement in increased physical activity. Mean time of physical activity in this group was sufficient to achieve additional health benefits in accordance with WHO recommendations.

Participation in analysed programmes, of both 2 weeks and 3 months duration, significantly improved health indices and physical fitness, cumulatively decreasing the risk of premature death. This should allow reaching lower risk of CVD. This finding is consistent with other research, mainly cross-sectional [6, 21, 44].

It seems that one would expect a greater reduction in risk of CVD when multidimensional programmes were more effective in reducing body fat mass, due to the presence of a moderately strong correlation between somatic indices (BMI and percentage of body fat) and a number of health indicators [45].

### 4.1. Limitations

The study has some limitation that have to be addressed: nutrition habits, smoking habits, medication, and physical activity due to leisure activities can affect the way how the health indices respond to the increased physical activity and should be recognised as potential biases. To decrease possible influences of nutrition habits changes, the participants were instructed to maintain their nutritional habits during the intervention programme.

## 5. Conclusion

The findings of this study showed that implementation of the OPA programme had a positive impact on somatic features, exercise capacity, biochemical indices, and risk of death from CVD. The presented programme can be regarded as an effective element of primary prevention of cardiovascular diseases among women older than 55 years.

## Figures and Tables

**Table 1:** Baseline subjects' characteristics (mean ± SD); *n* = 35.

Age [years]	65.4 ± 7.3
Body height [cm]	159.7 ± 5.60
Body mass [kg]	69.1 ± 12.4
BMI	26.7 ± 4.7
Fat mass [%]	37.4 ± 6.4
Waist circumference [cm]	89.9 ± 11.9
Hip circumference [cm]	105.9 ± 8.8
WHR	0.85 ± 0.07

Systolic blood pressure [mmHg]	133.2 ± 16.7
Diastolic blood pressure [mmHg]	78.0 ± 8.0
Heart rate [beats/min]	83.1 ± 10.9

FVC [L]	2.85 ± 0.57
FVC [%]	113.8 ± 16.2
FEV1 [L]	2.21 ± 0.42
FEV [%]	106.4 ± 16.7
Tiffeneau index [—]	0.78 ± 0.07
Tiffeneau index [%]	101.8 ± 9.3

Glucose [mmol/L]	5.24 ± 0.96
Total cholesterol [mmol/L]	5.60 ± 1.15
HDL cholesterol [mmol/L]	1.66 ± 0.38
LDL cholesterol [mmol/L]	3.46 ± 1.12
Triglycerides [mmol/L]	0.94 ± 0.43
hsCRP [mmol/L]	0.30 ± 0.36

Duration of test exercise [s]	294.4 ± 77.9
Maximal tolerated load [MET]	3.8 ± 0.5
Maximal tolerated load [W]	58.1 ± 12.2
10-year absolute CVD risk score [%]	5.3 ± 2.9

Note: WHR: waist to hip ratio; FVC: forced vital capacity; FEV1: forced expiratory volume in 1 second; FEV1/FVC: Tiffeneau-Pinelli index; TG: triglycerides; TC: total cholesterol; HDL-C: cholesterol high-density lipoprotein; LDL-C: cholesterol low-density lipoprotein; hsCRP: high sensitive C-reactive protein; CVD: cardiovascular diseases.

**Table 2:** Organization of the study.

Stage	Duration	Number of participants	Measurements
Baseline measurements	2 days	35	Body mass and composition Waist and hip circumferences Physical fitness Blood profile Blood pressure and heart rate Pulmonary indices CVD risk score

Group-based physical activity camp in a remote location (ALTP)	14 days	35	

Follow-up measurements I	2 days	29	Body mass and composition Waist and hip circumferences Physical fitness Blood profile Blood pressure and heart rate Pulmonary indices CVD risk score

Prevent Falls in the Elderly Programme (PFEP)	3 months	31	

Follow-up measurements II	2 days	23	Body mass and composition Physical fitness Blood profile Blood pressure and heart rate Pulmonary indices CVD risk score

Self-guided physical activities	18 months	26	

Follow-up measurements III	1 day	26	Physical Activity Survey
